# Non-Invasive Continuous Respiratory Monitoring on General Hospital Wards: A Systematic Review

**DOI:** 10.1371/journal.pone.0144626

**Published:** 2015-12-14

**Authors:** Kim van Loon, Bas van Zaane, Els J. Bosch, Cor J. Kalkman, Linda M. Peelen

**Affiliations:** 1 Division of Anesthesiology, Intensive Care and Emergency Medicine, University Medical Center Utrecht, Utrecht, The Netherlands; 2 Julius Center for Health Sciences and Primary Care and Division of Anesthesiology, Intensive Care and Emergency Medicine, University Medical Center Utrecht, Utrecht, The Netherlands; University of Tübingen, GERMANY

## Abstract

**Background:**

Failure to recognize acute deterioration in hospitalized patients may contribute to cardiopulmonary arrest, unscheduled intensive care unit admission and increased mortality.

**Purpose:**

In this systematic review we aimed to determine whether continuous non-invasive respiratory monitoring improves early diagnosis of patient deterioration and reduces critical incidents on hospital wards.

**Data Sources:**

Studies were retrieved from Medline, Embase, CINAHL, and the Cochrane library, searched from 1970 till October 25, 2014.

**Study Selection:**

Electronic databases were searched using keywords and corresponding synonyms ‘ward’, ‘continuous’, ‘monitoring’ and ‘respiration’. Pediatric, fetal and animal studies were excluded.

**Data Extraction:**

Since no validated tool is currently available for diagnostic or intervention studies with continuous monitoring, methodological quality was assessed with a modified tool based on modified STARD, CONSORT, and TREND statements.

**Data Synthesis:**

Six intervention and five diagnostic studies were included, evaluating the use of eight different devices for continuous respiratory monitoring. Quantitative data synthesis was not possible because intervention, study design and outcomes differed considerably between studies. Outcomes estimates for the intervention studies ranged from RR 0.14 (0.03, 0.64) for cardiopulmonary resuscitation to RR 1.00 (0.41, 2.35) for unplanned ICU admission after introduction of continuous respiratory monitoring,

**Limitations:**

The methodological quality of most studies was moderate, e.g. ‘before-after’ designs, incomplete reporting of primary outcomes, and incomplete clinical implementation of the monitoring system.

**Conclusions:**

Based on the findings of this systematic review, implementation of routine continuous non-invasive respiratory monitoring on general hospital wards cannot yet be advocated as results are inconclusive, and methodological quality of the studies needs improvement. Future research in this area should focus on technology explicitly suitable for low care settings and tailored alarm and treatment algorithms.

## Introduction

In high care facilities, like an Intensive Care Unit (ICU), monitors are often continuously reporting physiological patient variables, such as heart rate or respiratory rate. Physicians interpret these values and the resulting alarms, and act appropriately if needed to prevent physiological decline.

In contrast, on general hospital wards, patient monitoring takes place at a minimal level and merely consists of intermittent observations by nurses. Physiological variables are typically registered only once every shift, i.e., at best once every 8–10 hours.[1,2] Deterioration that occurs in between these observations is more likely to go unnoticed and could result in detrimental outcomes, such as cardiopulmonary arrest, unscheduled intensive care unit (ICU) admission, and in-hospital mortality.[1,3–5]

Since respiratory failure is the most common primary cause of ICU admission from general hospital wards [5–7], an abnormal respiratory rate (generally above 30 breaths per minute [1,8]) could be an important and sensitive clinical predictor for current or future serious adverse events.[4,9,10] In daily practice however respiration is only intermittently observed by caregivers and expressed as an approximate respiratory rate, sometimes combined with rough estimates of tidal volume (‘deep’, ‘shallow’), intermittent pulse oximetry, or more subjective parameters such as the patient’s skin color including apparent signs of cyanosis. Preferably such intermittent subjective observations are supported by reliable electronic or mechanical bedside equipment.[11]

The purpose of this systematic review was to examine whether *continuous* respiratory monitoring can assist caregivers on the general hospital ward with early detection of deteriorating patients, and improve patient safety by reducing the incidence of critical events. We reviewed publications on the use of continuous non-invasive respiratory monitoring in patients admitted to a general hospital ward in clinical practice. These publications include intervention studies, investigating the effect of continuous respiratory monitoring on clinical outcomes, and diagnostic studies, evaluating the ability to detect abnormal vital signs in comparison to a chosen reference standard. Furthermore, as methodological quality assessment tools for this type of research are currently unavailable, we summarize methodological pitfalls in the design and reporting of monitoring studies based on our findings in this systematic review.

## Materials and Methods

### Search strategy and study selection

Electronic databases Medline (Pubmed), Embase, CINAHL, and the Cochrane library were searched for publications on continuous respiratory monitoring on general hospital wards from 1966 through October 2014. Synonyms for ‘hospital ward’, ‘continuous monitoring’ and ‘respiration’ were included along with search filters (S1 File). Duplicates were automatically eliminated. Based on titles and abstracts, two reviewers (KL and EB) eliminated studies that did not evaluate a continuous non-invasive monitor, pediatric-, fetal- or animal studies, and studies that did not monitor a respiratory physiological variable. As respiration comprises ventilation and oxygenation, devices monitoring any of these vital signs were considered respiratory monitors. To find additional papers that were missed with our original search we also searched Web of Knowledge (URL: http://apps.webofknowledge.com). Since we aimed to focus on continuous respiratory monitoring in clinical practice, we included all original studies performed on general hospital wards, with the outcome defined as serious adverse events (mortality, cardiopulmonary arrests, ICU admission, and length of ICU or hospital admission). Furthermore, we also included diagnostic accuracy studies, which evaluated whether the device is able to detect respiratory abnormalities accurately.

### Data extraction

After selection of relevant articles, two reviewers (KL and EB) assessed full text articles independently. Two data extraction forms were developed, one for papers concerning clinical outcomes, one for articles concerning diagnostic accuracy (S2 File). Discrepancies between reviewers were discussed with an epidemiologist (LP) or a clinician (BZ) until consensus was reached among authors.

### Quality assessment

Two independent reviewers (KL and EB) assessed the methodological quality of included studies. Since there are no specific methodological quality assessment tools for research on monitoring technology, we used modified Standards for Reporting Studies of Diagnostic Accuracy (STARD) and Quality Assessment of Diagnostic Accuracy Studies (QUADAS) criteria for diagnostic studies, and the Consolidated Standards Of Reporting Trials (CONSORT) and Transparent Reporting of Evaluations with Nonrandomized Designs (TREND) statements for articles focusing on clinical outcomes.[12–15] These modified criteria are listed in the S3 File.

## Results

**Fig 1:**
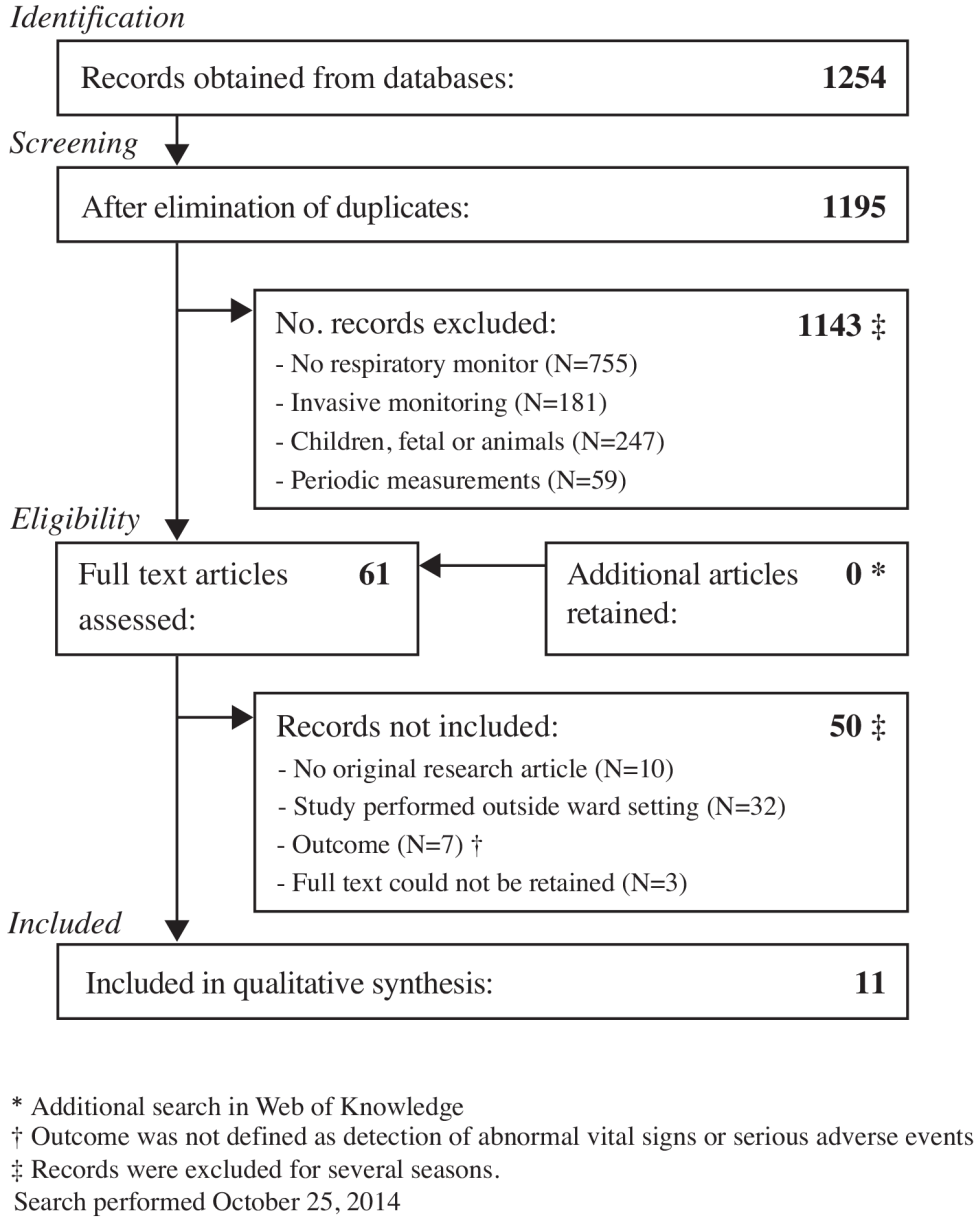
Flow diagram according to the PRISMA statement.

The search yielded 1254 publications, which were handled as presented in the flowchart (Fig 1). After reviewing titles and abstracts, 61 publications remained. From these, eleven studies were further analyzed; the other 50 studies were excluded as the paper did not describe original research (n = 10), the study was performed outside the general ward (n = 32), the study evaluated the agreement between two monitoring methods in healthy volunteers or did not investigate the monitoring system itself but used it as a tool to describe the population (n = 7), or full-text could not be retrieved (n = 3). The eleven studies that were included evaluated eight different monitoring systems.

The remainder of the results section below describes the six intervention studies from three different viewpoints.[16–21] An overview of the five diagnostic studies can be found in the supporting information (S1–S3 Tables).[22–26] First we describe the technical aspects of the monitoring systems (including the sensing principles, signal analysis, and caregiver notification), second we describe the study design, and third we summarize the individual study outcomes.

### I—Technical aspects of monitoring systems

Continuous electronic patient monitoring aims to translate physiological parameters that describe the current status of the patient’s vital systems into interpretable indicators to allow follow-up and appropriate action. The entire process consists of three steps: sensing the relevant parameter(s), interpretation of the measured variables (signal analysis), and notification of caregivers.[27] Table 1 gives an overview of these three aspects for each of the studies. For the diagnostic studies this can be found in S1 Table.

**Table 1:** Technical characteristics of non-invasive continuous monitors in intervention studies.

		Sensor technology					Signal analysis	Caregiver notification
Authors	Device	Respiratory parameters monitored	Sensing principle		Signal transmission	Detection of upper airway obstruction	Smart alarm control	Alarms displayed
**Brown** [16]	Early Sense piezoelectric sensor	BR	Chest wall movement through stretch	HR	Wireless	-	-	B, NB, C
**Hravnak** [17]	Biosign algorithm	BR, SpO2	Chest wall impedance, pulse oximetry	HR, BP	Hard-wired	-	+	B, C
**Kisner** [18]	Auricall pulse oximeter	SpO2	Pulse oximetry	HR	Wireless	-	-	NB, doctor’s message
**Ochroch** [19]	Nellcor pulse oximeter	SpO2	Pulse oximetry	-	Hard-wired	-	-	B, C
**Taenzer** [20]	Masimo pulse oximeter	SpO2	Pulse oximetry	HR	Hard-wired	-	-	NB
**Watkinson** [21]	Biosign algorithm	BR, SpO2	Chest wall impedance, pulse oximetry	HR, BP, T	Hard-wired	-	+	B

Hemoglobin oxygen saturation (SpO2), Breathing rate (BR), Heart rate (HR), Blood pressure (BP), Temperature (T), Nurse beeper (NB), Bedside (B), Central nursing station (C).

Smart alarm control: (-) none, (+) advanced data modeling techniques as fuzzy logic, neural networking, and pattern recognition to ease interpretation.

#### Sensing principles

A sensor translates vital signs such as respiratory rate or arterial oxygen saturation to an electronic signal. Monitors selected in this review measured breathing rate[16], oxygen saturation (SpO2)[18–20] or both[17,21]. Most devices incorporated conventional sensing principles, such as pulse oximetry to measure arterial oxygen saturation and electrocardiography (ECG) -based transthoracic impedance to measure respiratory rate.[17–21] The transmission of the signals from the patient to the device was wireless in two devices.[16,18] None of the devices evaluated in the intervention studies incorporated a sensing principle that is capable to directly identify upper airway obstruction by detecting loss of airflow.

#### Signal analysis

There was considerable variation in the way the crude electronic signals were handled and translated into interpretable numerical and graphical indicators. Four monitors used a simple translation. The other two monitors made use of so-called ‘smart alarm control’[17,21], where advanced data modeling techniques, such as fuzzy logic, neural networking, and pattern recognition [27,28], were used to ease interpretation of changes in multiple monitor signals and to reduce the amount of false alarms. The Biosign algorithm, which was investigated in the studies by Hravnak and Watkinson[17,21], involved conventional hardwired sensing principles, with new data driven algorithms to present a single score for the patient’s vital status. The signal analysis in the article of *Taenzer* was supplemented with efforts to reduce false positive alarms, including evaluation of the robustness of the arterial pulse wave, by calibration and by notification delay.[20]

#### Caregiver notification system

As ward nurses spend only a limited amount of their time in the immediate vicinity of the patient (the nurse to patient ratio varied between 1:4 and 1:10 patients in the studies), the monitoring system needs to alert caregivers in time to successfully facilitate prevention of serious adverse events. The simplest notification strategy used was a bedside audible and displayed alarm. In three studies, the monitor was able to alert responsible caregivers who were physically present in the patient’s room or near the central nursing station when an alarm was triggered.[17,19,21] The most extensive strategy was implemented by *Kisner* notifying physicians and nurses via mobile phone, where the physician received electronic documentation from the system describing the event [18].

### II Study design

Table 2 summarizes methodological quality of the intervention studies that were included. For the five diagnostic studies, this summary is provided in S2 Table. The six intervention studies aimed to evaluate the additional value of continuous bedside monitoring compared to intermittent physiological observations on clinical outcomes. *Watkinson* and *Ochroch* conducted randomized controlled trials.[19,21] In the comparator arm of the Watkinson trial patients’ vital signs were recorded manually and with electronic devices at intervals. For patients in the comparator arm of the trial by *Ochroch*, vital signs were measured and recorded intermittently and in more than 95% of the patients ECG telemetry monitoring was also performed.[19] Other studies used a before-after design[16–18,20], which has the drawback that differences in outcomes can also be due to differences in patient characteristics rather than just the intervention of continuous monitoring itself. This may in particular be the case in the study of *Kisner* and *Brown* in which also retrospective data was used.[16,18] *Brown* described that the decision on admission to the control- or intervention unit was practically random; however patient outcomes in the intervention unit before implementation of the monitor system, and the control unit before and after implementation differ.[16] In contrast to other studies, the before- after- study of *Taenzer* does present baseline characteristics only on the ward level rather than on the level of individual patients.

**Table 2:** Summary of methodological quality of the intervention studies.

Quality items	Brown [16]	Hravnak [17]	Kisner [18]	Ochroch [19]	Taenzer [20]	Watkinson [21]
**Representative sample**	+	+	+	+	+	+
**Randomized controlled trial**	-	-	-	+	-	+
**Predefined outcome measures**	+	+	+	+	-	+
**Adequate intervention description**	+	-	+	+	+	-
**Blinding of those assessing the outcome**	-	+	?	+	?	-
**Intention to treat**	?	-	?	?	-	+
**Demographic Characteristics**	+	+	+	+	-	-
**Report results for each study condition**	+	-	+	-	+	+
**Withdrawals explained**	-	-	-	+	-	+
**Limitations, generalizability discussed**	+	-	-	+	+	+

Quality items are described in detail in the supporting material (S3 File).

### III Outcomes of the studies

Table 3 presents the characteristics and outcomes for the intervention studies. In S3 Table the results for the diagnostic studies are summarized.

**Table 3:** Summary of characteristics and outcomes for intervention studies.

							Outcomes: control ~ intervention				
	Year	No. of patients	Mean age (year)	Ward	Nurse: patient ratio	Monitoring intensity *	ICU	SAE	LOS ICU	Patient comfort †	Alarm/ False alarm rate
**Brown**[16]	2014	3747 II	50	Medical Surgical	1:5	Surveillance	2.7% ~ **2.6%** P = .91 ‡	0.63% ~ **0.09%** P = .01 ‡	120.1 ~ **63.4** days/1000 P = .10	+	0.03 alerts per day per bed
**Hravnak**[17]	2011	631	57	Surgical	1:4–8	Surveillance	-	20% ~ **15%** P = .09	-	-	1.01 alerts per patient day ‡
**Kisner**[18]	2009	357	64	Cardiac	?	Conditional	-	28% ~ **18%** P = .06	-	+	-
**Ochroch**[19]	2006	1214	61	Surgical Cardiac	?	Surveillance	8.5% ~ **6.7%** P = .33	-	-	-	-
**Taenzer**[20]	2010	5959 ¶	57	Surgical Medical	1:5	Surveillance	2.1% ~ **1.1%** P = .003 ‡	0.34% ~ **0.12%** P = .01	-	+	4 alerts per patient day
**Watkinson**[21]	2006	402	73	Medical Surgical	1:6–10	Conditional	-	58% ~ **56%** P = .76	-	-	2.93 alerts per patient day ‡

* Monitoring intensity is defined as surveillance when patients were monitored for 100% during their hospitalization, or conditional in case of specific patient categories for a limited period.

† Patient comfort: (+) good patient comfort as derived from the study results or reviewers’ rating.

‡ Outcome measures and alarm rates were calculated with extracted information from the paper.

§ Statistics computed for detection of critical events with respiratory and heart rate alerts with retrospective (post hoc) determined optimal thresholds.

II 7643 patients were studied, but in this summary we use only data from the intervention unit.

¶ Number of patient discharges.

ICU = Intensive care unit, LOS = length of stay, SAE = serious adverse event as defined by the original paper.

The monitoring systems in the articles of *Hravnak* and *Watkinson* are identical.

**Fig 2:**
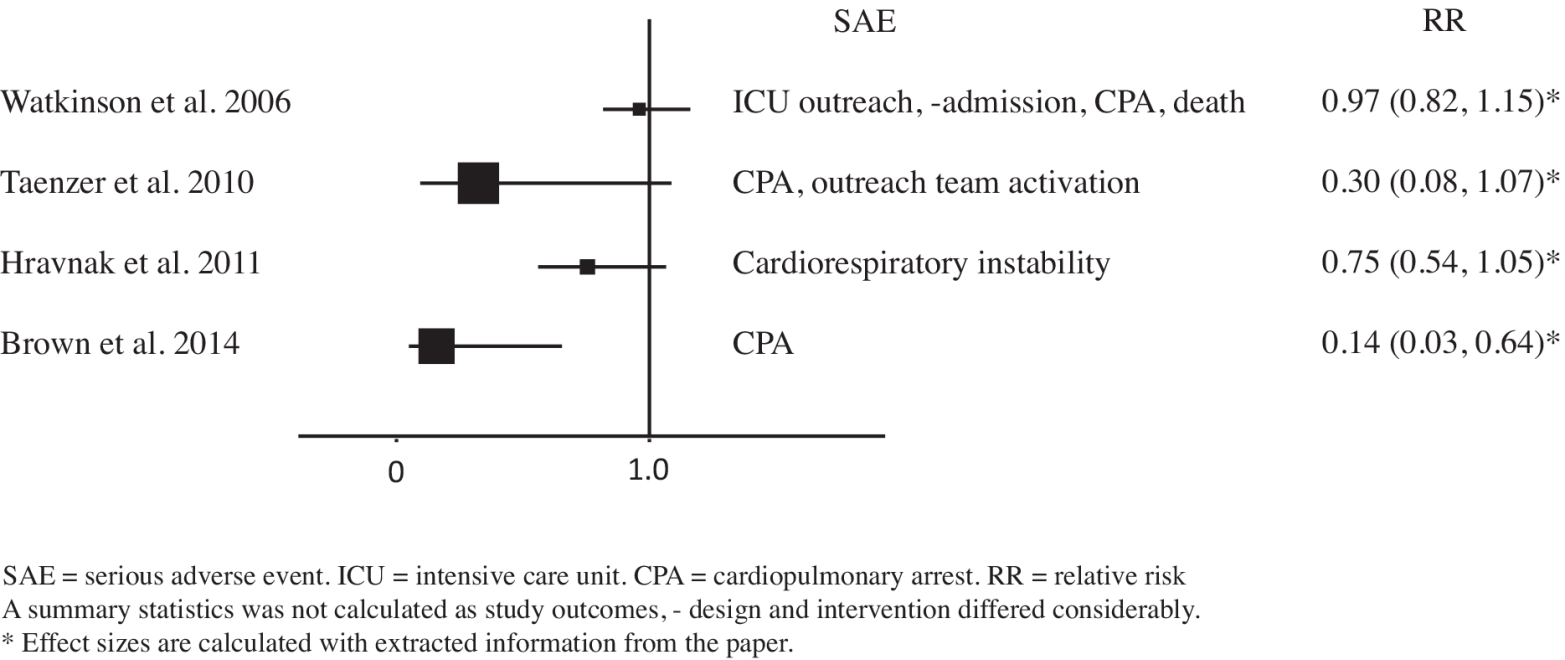
Forest plot of comparison: Serious Adverse Events (SAE) for continuous versus intermittent respiratory monitoring on general hospital wards.

**Fig 3:**
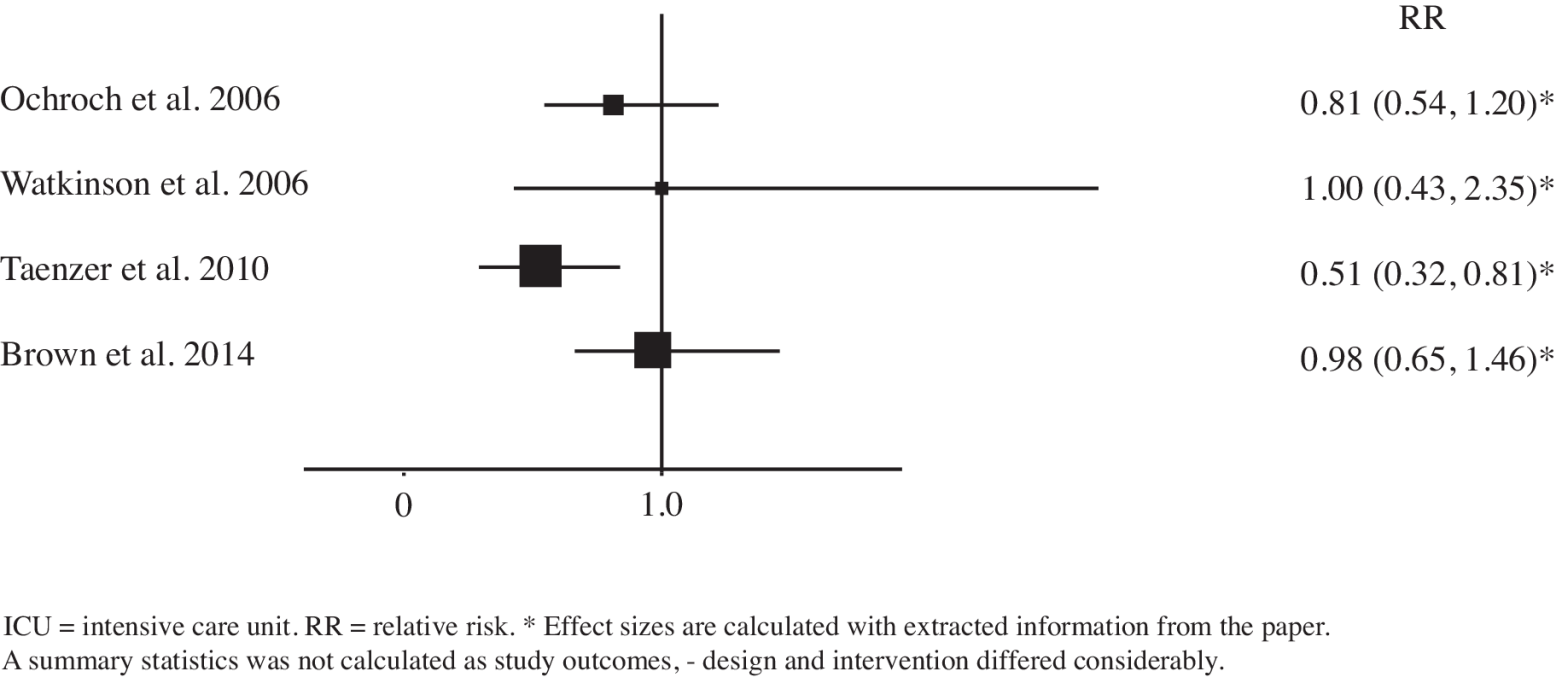
Forest plot of comparison: ICU admission for continuous versus intermittent respiratory monitoring on general hospital wards.

In all intervention studies the incidence of the primary outcome was lower during continuous monitoring, although not always statistically significant. *Taenzer* found a statistically significant reduction of ICU admission rates (2.1% vs. 1.1%, p = 0.003).[20] In the *Brown* study, the length of stay on the ICU decreased significantly after implementation of the monitoring system when compared to the control unit and the intervention unit before implementation (respectively 63.4 days vs. 85.4 days vs. 120.1 days per 1000 patients).[16] When we compare the length of stay on the ICU for patients admitted to the intervention unit, the decrease after introduction of the monitor system did not reach statistical significance. Overall, the ICU admission rates in this study did not change.[16] In Table 3 outcomes are reported as proportions and to facilitate comparison between before-after studies only data from the intervention unit was summarized. Relative risks (RRs) were calculated with information extracted from the papers for serious adverse events (SAE) and ICU admission rates (Figs 2 and 3). RRs for SAEs ranged from 0.14 to 0.97 and RRs for ICU admission ranged from 0.51 to 1.00. We do not provide pooled summary estimates in the forest plots, as studies differed considerably in terms of outcome, intervention and study design. Three studies chose a composite endpoint as primary outcome,[17,20,21] combining serious adverse events defined as ICU admission, readmission, rescue events and mortality. *Kisner* chose an intermediate outcome, the incidence of atrial fibrillation, which is known to be associated with increased morbidity, mortality, prolonged hospital stay and increased costs after cardiothoracic surgery.[18] The primary outcome of *Ochroch* was defined as ICU admission rate.[19] Three outcomes were mentioned as primary outcomes.

Patient comfort was reportedly minimally affected by the monitor system in the study of *Taenzer*, *Kisner* and *Brown*.[16,18,20] The acceptance rate of the Masimo pulse oximeter was high with only 1.8% of patients refusing to wear the sensor continuously.[20] 16% of the patients in the study of *Watkinson*, in which conventional sensor technology was used, were monitored for the full 72 h.(21) The monitor was removed if requested by nurses to allow mobilization (37%), by the patient (30%), in case of a serious adverse event (18%), or for other reasons (15%). In the study of *Kisner* the pulse oximeter was wireless and placed on the patient’s ear lobe.[18] None of the selected articles mentioned the occurrence of pressure ulcers during or after long-term vital sign monitoring.

Alarm rates were mentioned in four studies and ranged from 0.03 to 4 alerts per patient or bed per day.[16,17,20,21]

## Discussion

Based on the findings of this systematic review, implementation of routine continuous respiratory monitoring on general hospital wards cannot yet be advocated. Results were inconclusive and in the available intervention studies clinical implementation was incomplete. Furthermore, methodological quality of most studies was moderate, e.g. ‘before-after’ designs, incomplete reporting of primary outcomes, and incomplete clinical implementation of the monitoring system.

A decade ago, *Folke* reported on available techniques and devices for non-invasive respiratory monitoring.[11] At that time, clinical research evaluating these devices was not available. Our systematic review focused on this next step, by including articles evaluating the clinical impact of non-invasive continuous respiratory monitoring systems. It is interesting to note that we did not observe such a large diversity in sensing principles as described earlier by *Folke*.[11] Our search strategy yielded only five diagnostic and six intervention studies that evaluated continuous respiratory monitors on general hospital wards. The selected articles predominantly applied conventional sensing principles sometimes combined with innovative signal analysis to monitor general ward patients. In the present review, only two monitoring devices covered sensing principles that were tailored to low care clinical settings, including patient comfort demands.[16,25,26] Conventional sensing principles, often leaving the patient attached to wires, give less patient comfort and more frequent dislodgement of the sensor. A high number of false positive alarms, e.g. by sensor dislodgement, can be extremely disruptive for patient and caregiver, especially in low care clinical settings.[29,30] The frequency and tolerability of false positive alarms for each unique monitoring system in this setting was not extensively studied for the monitoring systems that were included in our systematic review.

This systematic review on continuous respiratory monitoring has some limitations. First, we were unable to pool study results into one overall effect estimate, as the studies differed considerably with respect to the physiological variables that were monitored. Devices monitoring any vital sign related to ventilation or oxygenation matched the inclusion criteria, but with these devices many other vital parameters (i.e. heart rate, temperature) were included in the monitoring strategy under study. A second reason for refraining from pooling individual effect estimates were differences in study design and methodological quality of the studies. A second limitation of this systematic review is the domain we chose in the search and selection strategy. By only selecting studies that were performed on hospital wards, we excluded devices that were potentially suitable for this setting but have not yet been studied as such. Monitoring devices can ‘legally’ be used in a low care patient ward setting if they were developed and validated in an ICU or operating room setting. At the moment such ‘off label’ use is generally accepted, but hardly investigated. Therefore, this systematic review is not able to give an overview of all sensor technologies and monitoring devices that are actually used in the low care clinical setting. Finally, our review almost certainly suffers from publication bias. Regulations regarding the need for clinical validation research before introducing a new non-invasive monitoring device on the market are less stringent when compared to pharmaceutical interventions and it is conceivable that device manufacturers will not support publication of negative study results.

A solid methodological base for research on physiological monitoring is currently scarce and methodological quality of the included studies was moderate to poor. This impedes the assessment of the relevance and validity and makes comparison of alternative monitoring strategies difficult. Based on our findings, we provide the following suggestions for the design and reporting of the evaluation and validation of continuous monitoring systems:

Continuous physiological monitoring is a process of repeatedly sensing one or more physiological variables, analyzing the resulting time series, followed by notification and action on pre-defined changes in relevant vital signs. The ideal monitoring strategy should include unobtrusive sensors that are well tolerated by the patient, have a high sensitivity and a high positive predictive value. The passive sensor array in the *Jacobs* study had a high positive predictive value of 98.9% with a corresponding low false alarm rate of 0.16 per patient day.[25] The latter is important to alert caregivers in time, but at the same time prevent ‘alarm fatigue’ resulting from false alarms.[31,32] Finally, a well-designed notification system might also contain decision support algorithms to help caregivers decide on the most appropriate immediate therapeutic interventions.

In monitoring studies the elements of the monitoring strategy should preferably be investigated separately, and subsequently as a whole. To be able to study the effect on patient outcome, the entire monitoring strategy, from sensing to action, should be operational. A single dysfunctional component can completely obscure potential improvement of patient outcome. A clear example of the latter is the carefully designed and reported randomized controlled trial by *Watkinson*, where continuous monitoring was not found to influence the incidence of ICU admission and cardiac arrest.[21] As the authors indicate themselves, this might be explained by the fact that they were unable to implement timely caregiver notification of abnormal vital signs during the trial. As a result, abnormalities often went unnoticed, and no action was undertaken to prevent further deterioration. Ideally, after optimizing the individual components e.g. the notification strategy, the clinical effect of continuous monitoring on patient outcomes is studied as a complete monitoring strategy in a randomized controlled trial. Note that this includes consensus on the therapeutic interventions performed. By not doing so, and thus performing various interventions for the same incidents, one does not test the monitoring strategy, but rather the interventions performed. Clinical evaluation of monitoring strategies shows parallels with the workup for a new diagnostic tool, starting with initial safety, diagnostic accuracy (test-research), and reproducibility studies. If a monitor passes these initial tests, subsequent studies should focus on the additional diagnostic value, and eventually diagnostic intervention research investigating the effect on patient outcomes. [33–35]

In monitoring studies, selection and reporting of the outcome should be done carefully. In contrast to a ‘classic’ diagnostic workup, monitoring does not estimate the probability of the presence of a disease once. Instead, the monitoring system tries to continuously update the probability that a patient will become critically ill in the near future. This difference brings along several issues in the selection and definition of the outcome measure.

First, ‘vital instability’ is not a diagnosis per se, but an undesirable patient status which may lead to critical illness and potential death in the very near future. Hence, its definition implicitly contains elements of the prognosis of the patient. For example, passing a given threshold for a vital sign (heart rate, respiratory rate, oxygen saturation or systolic blood pressure) makes the patient ‘instable’ as it is known from literature that passing this threshold strongly increases the probability of a serious adverse event from 2.5% to 35%.[36]

Second, vital signs change over time as part of natural history of a disease. Vital instability is hence a gliding scale, which may be interpreted differently by different caregivers. Hence, clear-cut and detailed definitions are required, e.g., the maximum acceptable deviation from baseline in percentages (%).

Third, when comparing the measurements by the monitoring system with a reference standard, differences in measurement frequency are encountered: the monitoring system senses continuously, whereas the reference standard are observations at discrete time points, for example once per shift by the nurse. When calculating accuracy the repeated measurements from the monitoring system have to be ‘summarized’ into a binary outcome. The way this summary is made influences the conclusion on the accuracy of the monitoring systems.

Fourth, selection of the clinical outcome in the diagnostic intervention study of a monitoring strategy is not straightforward. Mortality would seem a logical primary outcome in an intervention study evaluating the effect of safety monitoring. However, in-hospital mortality rates are low (between 0.8% for elective and 5.5% for emergency admissions [37]) and many hospital deaths cannot be prevented as they are due to the natural progression of disease. Unplanned ICU admission [1,36,38–40] and cardiopulmonary arrest rates [3,40,41] were often chosen in publications evaluating implementation of early warning systems. However, unplanned admission to the ICU might be considered a serious adverse event but alternatively, it can be an adequate early therapeutic decision that will positively influence the course of disease. Therefore, rescue events including unplanned ICU admissions should be explicitly predefined and considered as composite endpoints. An alternative substitute for mortality could be the length of hospital or ICU stay as in the study of *Brown*.

A final recommendation for monitoring research is to focus not only on patient outcomes, but also on feasibility of (part of) the monitoring strategy. This includes patient acceptability (freedom of movement, possible discomfort) and acceptability for nursing staff (ease of interpretation, false alarm rate, notification system–timely appropriate decision support versus disruptive irrelevant alerts). For example, future miniaturized wireless sensor technology may possibly eliminate the drawbacks of conventional ‘wired’ monitoring such as patient discomfort and increased staff workload from false alarms caused by sensor dislodgment. Ignoring aspects of feasibility may obscure the additional value of an otherwise well designed monitoring system.

## Conclusions

Based on the results of the studies selected in this systematic review, implementation of routine continuous (respiratory) monitoring on general hospital wards cannot yet be advocated. The methodological quality of studies evaluating electronic patient monitors on general hospital wards needs improvement. Future research in this area should focus on technology explicitly suitable for low care clinical settings and development of alarm algorithms tailored to the specific hospital ward setting.

## Supporting Information

S1 File Search strategy. Search was performed on October 25, 2014.
(PDF)

S2 File Checklist for intervention research and diagnostic studies. Data extraction form.
(PDF)

S3 File Modified assessment tool used to describe study quality. Since there are no specific methodological quality assessment tools for research on monitoring technology, we used modified Standards for Reporting Studies of Diagnostic Accuracy (STARD), Quality Assessment of Diagnostic Accuracy Studies (QUADAS), Consolidated Standards Of Reporting Trials (CONSORT) and Transparent Reporting of Evaluations with Nonrandomized Designs (TREND) statements.
(PDF)

S4 File References (50 articles) that were not included in the systematic review. (PDF)

S5 File Prisma 2009 checklist. (PDF)

S1 Table Technical characteristics of non-invasive continuous monitors in diagnostic studies. (DOCX)

S2 Table Summary of methodological quality of the diagnostic studies. (DOCX)

S3 Table Summary of characteristics and outcomes for diagnostic studies. (DOCX)
