# Supplementary material for: Non-Invasive Continuous Respiratory Monitoring on General Hospital Wards: A Systematic Review
Source: PLoS One. 2015 Dec 14;10(12):e0144626. doi: 10.1371/journal.pone.0144626 (PMC4684230; doi:10.1371/journal.pone.0144626)
Supplement: S1 File — Search was performed on October 25, 2014. (PDF) [file pone.0144626.s001.pdf]

S1 File. Search performed on October 25, 2014

Database	Limits	Synonyms	Articles
PubMed	[Title/Abstract] Humans, English, Dutch	("Patients' rooms"[MeSH] OR "ward" OR "wards" OR "inpatient" OR "inpatients" OR "inhospital" OR "nursing" OR "hospitalized") AND ("Clinical Alarms"[Mesh] OR "Monitoring, Physiologic"[Mesh]) OR "monitoring" OR "monitor" OR "monitors" OR "alert system" OR "observation" OR "observations" OR "surveillance") AND ("continuous" OR "continuously" OR "continued" OR "permanent" OR "constant" OR "automated" OR "routine") AND ("Respiration"[MeSH] OR "respiratory" OR "respiration" OR "breathing" OR "oxygenation" OR "oximetry" OR "saturation" OR "ventilation" OR "ventilatory" OR "pulmonary" OR "capnography")	425
EMBASE	:ab,ti<br>Limits: Humans, English, Dutch, EMBASE	‘ward'/exp OR 'nursery'/exp OR 'hospital patient'/exp OR 'hospital'/exp OR 'ward':ti,ab AND ('breathing'/exp OR 'capnography'/exp OR 'oxygen saturation'/exp OR 'oximetry'/exp OR 'oxygenation'/exp OR 'lung ventilation'/exp OR 'respiration'/exp OR 'respiratory' OR 'ventilatory' OR 'pulmonary') AND ('continuous process'/exp OR 'continuous' OR 'continuously') AND ('monitoring'/exp OR 'patient monitoring'/exp OR 'monitor'/exp OR 'observation'/exp OR 'surveillance' OR 'monitoring')	820
CINAHL	Limits: AB Abstract, Humans, exclude Medline	AB (ward OR wards OR inhospital OR hospitalized) and AB (monitor OR monitoring OR alert system OR observation OR surveillance) and AB (continuous OR continuously OR automated OR routine OR continued) and (respiration OR respiratory OR breathing OR pulmonary OR ventilatory OR oxygenation OR oximetry OR ventilation OR capnography)	3
Cochrane	in Title, Abstract or Keywords, Cochrane Reviews	Patients' rooms OR ward OR wards OR inhospital OR inpatient OR inpatients OR hospitalized AND monitoring OR monitor OR monitors OR alert system OR observation OR clinical alarms OR observations OR surveillance AND continuous OR continuously OR continued OR permanent OR constant OR automated OR routine AND respiration OR respiratory OR breathing OR oxygenation OR oximetry OR saturation OR ventilation OR ventilatory OR pulmonary OR capnography	6
		total	**1254**
		after elimination of duplicates	**1195**
