# Supplementary material for: Non-Invasive Continuous Respiratory Monitoring on General Hospital Wards: A Systematic Review
Source: PLoS One. 2015 Dec 14;10(12):e0144626. doi: 10.1371/journal.pone.0144626 (PMC4684230; doi:10.1371/journal.pone.0144626)
Supplement: S2 File — Data extraction form. (PDF) [file pone.0144626.s002.pdf]

Checklist for Intervention research. Systematic Review Monitoring

Title …….

Author ……………………………………………….Device………………………………………………..

Journal, publication date …………………………………………………………………………….

Reviewer…………………………………………….Date…………………………………………………..

**Relevance**	Yes	No	Unclear
1. Is the spectrum of patients representative of a general hospital ward population?	( )	( )	( )
2. Provides the investigated device or system decision support, analyzing the signals to present state a patients' health?	( )	( )	( )
3. Is the monitoring device wireless?	( )	( )	( )
4. Is the monitoring device contactless?	( )	( )	( )
5. Is the test device easily applicable in low care clinical practice?	( )	( )	( )

Validity

Quality/ design

	Yes	No	Unclear
5. Randomized allocation?	( )	( )	( )
6. Sampling: Cohort / Case-Control / Case series / other ……………………………………….*			( )
7. Was the sample size calculation preformed?	( )	( )	( )
8. Was the method of recruitment/sampling systematically performed?	( )	( )	( )
9. Were primary and secondary outcome measures clearly predefined?	( )	( )	( )
10. Time span: How long was the intervention delivered? ……………………………………….			( )
11. Who assessed/ acted on the initial results from the monitor? ………………………………			( )
12. Who was blinded to the study condition assignment? Participants / care providers / those assessing outcomes *			( )
13. Statistical method to compare groups for outcomes: …………………………………………….			( )

Quality / study execution

	Yes	No	Unclear
14. Population, setting: ………………………………………………………………………………………			( )
15. When was the study performed (Date) ………………………………………………………………			( )
16. Participants. Baseline characteristics reported?	( )	( )	( )

	Patients	Age	Male			
Study group						
Usual care						

	Yes	No	Unclear
17. Index monitor, name: ………………………………………………………………………………………			( )
18. Method of respiratory observation (e.g. pulse oximetry): ………………………………………			( )
19. Measurement (e.g. humidity in expired air): …………………………………………………………			( )
20. Sensor placement (e.g. on the ear):……………………………………………………………………			( )
21. Additional vital sign measurements (e.g. HR):……………………………………………………			( )
22. Was standardized care applied at all times?	( )	( )	( )
23. Were any other health care changes made along with the introduction of the monitoring system?	( )	( )	( )
24. Primary endpoint …………………………………………………………………………………………			( )
Secondary endpoints ……………………………………………………………………………………			( )
25. Was the flow of participants through each stage clear: enrollment, assignment, allocation, intervention, follow-up?	( )	( )	( )
Enrollment: …………………… (no. participants screened for eligibility)			( )
Assignment: …………………… (no. participants assigned to a condition)			( )
Intervention exposure: ….. (no. participants receiving each intervention)			( )
Follow-up ……………………… (no. participants who completed follow-up)			( )

** delete what is not applicable*

		Yes	No	Unclear
26.	Was a summary of results provided for each primary- and secondary outcome?	( )	( )	( )
27.	Mortality			( )
28.	ICU admission			( )
29.	Length of hospital stay			( )
30.	Length of ICU stay			( )
31.	Cardiopulmonary arrests			( )
32.	Other:..			( )

Quality / article

		Yes	No	Unclear
33.	Is the research question clearly stated?	( )	( )	( )
34.	Is the target population and study sample clearly described?	( )	( )	( )
35.	Is the intervention clearly described to allow replication?	( )	( )	( )
36.	Is the data acquisition described sufficiently?	( )	( )	( )
37.	Were the results/ outcomes clearly and correctly reported?	( )	( )	( )
38.	Were the indeterminate results (artifacts) and outliers reported?	( )	( )	( )
39.	Were the results discussed taking into account -the trial limitations?	( )	( )	( )
	-generalisability?	( )	( )	( )
	-benefit and harms?	( )	( )	( )

Checklist for diagnostic studies. Systematic Review Monitoring

Title ………

Author ………………………………………………Device……………………………………………….

Journal, publication date ……………………………………………………………………………

Reviewer……………………………………………Date………………………………………………….

**Relevance**		Yes	No	Unclear
1.	Is the spectrum of patients representative of a general hospital ward population?	( )	( )	( )
2.	Provides the investigated device or system decision support, analyzing the signals to present state a patients' health?	( )	( )	( )
3.	Is the monitoring device wireless?	( )	( )	( )
4.	Is the monitoring device contactless?	( )	( )	( )
5.	Is the test device easily applicable in low care clinical practice?	( )	( )	( )

Validity

Quality / design

		Yes	No	Unclear
6.	Diagnostic accuracy / comparing accuracy between tests / methods-comparison*			( )
7.	Sampling: Cohort / Case-Control / Case Series / other: ………………………………… *			( )
8.	Was a sample size calculation performed?	( )	( )	( )
9.	Was the method of recruitment/sampling performed systematically?	( )	( )	( )
10.	Were cutoff points (normal vs. abnormal) or categories of both reference test and investigated device determined previously?	( )	( )	( )
11.	Were the reference measurements performed simultaneously with the index measurements (cross-sectional)?	( )	( )	( )
12.	Blinding of the index monitor?	( )	( )	( )
13.	Blinding of the reference monitor?	( )	( )	( )

Quality/ study execution

		Yes	No	Unclear
14.	Population, setting: …………………………………………………………………………			( )
15.	When was the study performed (Date) ………………………………………………………			( )
16.	Participants, baseline characteristics reported?	( )	( )	( )

	Patients	Age	Male				
No. %							

		Yes	No	Unclear
17.	Index monitor, name: ………………………………………………………………………			( )
18.	Method of respiratory observation (e.g. pulse oximetry): ………………………………			( )
19.	Measurement (e.g. humidity in expired air): ………………………………………………			( )
20.	Sensor placement (e.g. on the ear):………………………………………………………			( )
21.	Additional vital sign measurements (e.g. HR):…………………………………………			( )
22.	Reference test:…………………………………………………………………………………			( )
23.	Was standardized care applied at all times?	( )	( )	( )
24.	Were any other health care changes made along with the introduction of the monitoring system?	( )	( )	( )
25.	Is a cross tabulation reported?	( )	( )	( )

	Ref+	Ref-
Test+		
Test-		

26. Is diagnostic accuracy and statistical uncertainty reported? ( ) ( ) ( )

Sens	Spec	PPV	NPV	LR+	LR-

27. If comparability between tests is tested: Is a Bland Altman analysis done? ( ) ( ) ( )

Mean	SD	-limit	+limit

Quality / article

28. Is the research question clearly stated?	( )	( )	( )
29. Is the target population and study sample clearly described?	( )	( )	( )
30. Is the index monitor clearly described?	( )	( )	( )
31. Is the reference monitor clearly described?	( )	( )	( )
32. Is the data acquisition described sufficiently?	( )	( )	( )
33. Were the results/ outcomes clearly and correctly reported?	( )	( )	( )
34. Were the indeterminate results (artifacts) and outliers reported?	( )	( )	( )
35. Is clinical applicability of study findings reported?	( )	( )	( )

** delete what is not applicable.*
