# Supplementary material for: Non-Invasive Continuous Respiratory Monitoring on General Hospital Wards: A Systematic Review
Source: PLoS One. 2015 Dec 14;10(12):e0144626. doi: 10.1371/journal.pone.0144626 (PMC4684230; doi:10.1371/journal.pone.0144626)
Supplement: S3 File — Since there are no specific methodological quality assessment tools for research on monitoring technology, we used modified Standards for Reporting Studies of Diagnostic Accuracy (STARD), Quality Assessment of Diagnostic Accuracy Studies (QUADAS), Consolidated Standards Of Reporting Trials (CONSORT) and Transparent Reporting of Evaluations with Nonrandomized Designs (TREND) statements. (PDF) [file pone.0144626.s003.pdf]

S3 File. Modified assessment tool to describe study quality

Title:	Author:	Reviewer:

	Diagnostic study	+/-	Intervention study	+/-
1.	Spectrum of patients was representative of target population (1.1)		Spectrum of patients representative of target population (4.1)	
2.	Patients sampling method is a consecutive series of participants (cohort) (2.5)		Method to assign units to study conditions was randomized allocation (3.3, 4.8)	
3.	Reference standard likely to classify target condition (1.3, 2.7)		Completely predefined primary and secondary outcome measures (3.6, 4.6)	
4.	Execution of the index test was described in sufficient detail to permit replication (1.8, 2.8, 2.9)		Sufficient reporting on interventions for each group to allow replication (3.5, 4.4)	
5.	Results from both index monitor and reference standard were interpreted separately (blinding) (1.10, 1.11, 2.11)		Those assessing the outcomes were blinded to study condition assignment (3.11a, 4.9)	
6.	The whole sample or a random selection of the sample received verification by a reference standard (1.5)		Analysis strategy was "Intention to treat" (3.16, 4.16)	
7.	Demographic characteristics of the study population (2.15)		Baseline characteristics for each study condition (3.15, 4.14)	
8.	Report estimates of diagnostic accuracy and measures of statistical uncertainty (2.21)		For each primary and secondary outcome results were summarized for each study condition (3.17a, 4.17)	
9.	Withdrawals from the study were explained (1.14, 2.16)		Lost to follow-up and exclusions after assignment were reported and explained (3.13b, 4.19)	
10.	Discusses the clinical applicability of the study findings (2.25)		Discusses limitations, generalizability, interpretation (3.20-22, 4.20-22)	

References with paragraphs from available methodological quality assessment tools:
1. QUADAS tool, 2. STARD checklist, 3. CONSORT checklist, 4. TREND statement.
