# Supplementary material for: Non-Invasive Continuous Respiratory Monitoring on General Hospital Wards: A Systematic Review
Source: PLoS One. 2015 Dec 14;10(12):e0144626. doi: 10.1371/journal.pone.0144626 (PMC4684230; doi:10.1371/journal.pone.0144626)
Supplement: S4 File — (PDF) [file pone.0144626.s004.pdf]

S4 File. References (50 articles) that were not included in the systematic review. "Non-invasive continuous respiratory monitoring on general hospital wards: A systematic review."

1. Banks J, McArthur J, Gordon G. Flexible monitoring in the management of patient care processes: a pilot study. Lippincotts Case Manag 2000;5(3):94,103; 104-6.
Reason not included= no full text available. Author request per email without reply.

2. Bellomo R, Ackerman M, Bailey M, Meale R, Clancy G, Danesh, et al. A controlled trial of electronic automated advisory vital signs monitoring in general hospital wards. Crit Care Med 2012; 40(8):2349-61.
Reason not included= although suspected, automated does not mean continuous monitoring.

3. Bowdle TA. Nocturnal arterial oxygen desaturation and episodic airway obstruction after ambulatory surgery. Anesth Analg 2004; 99(1):70-6.
Reason not included= outcome was not defined as abnormal vital signs/SAE, as the monitor was not subject of study.

4. Bowton DL, Scuderi PE, Harris L, Haponik EF. Pulse oximetry monitoring outside the intensive care unit: progress or problem? Ann Intern Med 1991; 115(6):450-4.
Reason not included= study performed outside the general hospital ward

5. Bradley BD, Green G, Ramsay T, Seely AJE. Impact of sedation and organ failure on continuous heart and respiratory rate variability monitoring in critically ill patients: A pilot study. Critical Care Medicine 2013;41(2):433-44
Reason not included= study performed outside the general hospital ward

6. Campana LM, George E, Freeman J, Ladd D, MacNabb C, Lee JC, Voscopoulos C. The use of noninvasive respiratory volume monitoring to assess obstructive sleep apnea in the post-anesthesia care unit. Anesthesia and Analgesia 2013;116(SUPPL1):
Reason not included= study performed outside the general hospital ward

7. Christensen M, Lie C, Rosenberg J. Continuous pulse oximetry in the general surgical ward: Nellcor N-200 versus Nellcor N-3000. Anaesthesia 1999 54(3):253-7.
Reason not included= outcome was not defined as abnormal vital signs/SAE, as the monitor was not subject of study. But the outcome was defined as the percentage of false alarms for different signal analysis algorithms.

8. Ciccone A, Celani MG, Chiaramonte R, Rossi C, Righetti E. Continuous versus intermittent physiological monitoring for acute stroke. Cochrane Library 2013.
Reason not included= study performed outside the general hospital ward

9. Delerme S, Freund Y, Renault R, Devilliers C, Castro S, Chopin S, et al. Concordance between capnography and capnia in adults admitted for acute dyspnea in an ED. Am J Emerg Med 2010;28(6):711-4.
Reason not included= study performed outside the general hospital ward

10. Di Rienzo M, Rizzo F, Meriggi P, Bordoni B, Brambilla G, Ferratini M, et al. Applications of a textile-based wearable system for vital signs monitoring. Conf Proc IEEE Eng Med Biol Soc 2006;1:2223-6.
Reason not included= not an original article, and was not performed on a general hospital ward but in healthy volunteers.

11. Egleston CV, Ben Aslam H, Lambert MA. Capnography for monitoring non-intubated spontaneously breathing patients in an emergency room setting. J Accid Emerg Med 1997;14(4):222-4.
Reason not included= study performed outside the general hospital ward

12. Elliott M, Tate R, Page K. Do clinicians know how to use pulse oximetry? A literature review and clinical implications. Aust Crit Care 2006;19(4):139-44.
Reason not included= not an original article.

13. Federico N, Federico T, Federico F, Sternik G, Taghizadeh F, Esmaeli-Azad B. Miniaturized wearable sensors for wireless realtime vital sign recordings cloud based data transfer storage and analysis. FASEB Journal 2014;28(1):
Reason not included= not an original article

14. Freeman J, Lalli M, Yocum N, Panasyuk A, Panasyuk S, Lew R. Non-invasive monitoring of tidal volume and minute ventilation in non-intubated patients. Critical Care Medicine 2011;39(SUPPL12):88

Reason not included= study performed outside the general hospital ward

15. Hall D, Gibson K, Morley D, Wylie M, Rae S. Increasing the effective use of capnography in recovery: A multi-faceted trainee-delivered approach to quality improvement in anaesthesia. Anaesthesia 2014;69(SUPPL3):14
Reason not included= study performed outside the general hospital ward

16. Heaney LM. Cardiac and respiratory monitoring of acute stroke patients. Heart Lung 1977;6(3):469-74.
Reason not included= study performed outside the general hospital ward
17. Janssens JP, Perrin E, Bennani I, De Muralt B, Titelion V, Picaud C. Is continuous transcutaneous monitoring of PCO2 (TcPCO2) over 8 h reliable in adults? Respir Med 2001;95(5):331-5
Reason not included= study performed outside the general hospital ward

18. Kelly A, Klim S. Agreement between arterial and transcutaneous PCO2 in patients undergoing non-invasive ventilation. Respir Med 2011; 105(2):226-9
Reason not included= study performed outside the general hospital ward

19. Krieger BP. Ventilatory pattern monitoring: Instrumentation and applications. Respir Care 1990; 35(7):697-708
Reason not included= study performed outside the general hospital ward

20. Krieger B. Feinerman D, Zaron A, Biszousky F. Continuous noninvasive monitoring of respiratory rate critically ill patients. Chest 1986; 90(5):632-4.
Reason not included= study performed outside the general hospital ward

21. LeGrand TS, Peters JI. Pulse oximetry: Advantages and pitfalls. J Respir Dis 1999;20(3):195-206.
Reason not included= not an original article.

22. Lim BL, Kelly AM. How useful is transcutaneous carbon dioxide monitoring in the adult emergency department?. Hong Kong J Emerg Med 2010;17(1):82-4.
Reason not included= study performed outside the general hospital ward

23. MacNabb CM, Brayanov J, Freeman J, George E. Developing a protocol utilizing non-invasive respiratory volume monitoring to identify patients at risk for opioid induced respiratory depression. Circulation 2013;128(22):
Reason not included= not an original article, and the study was not performed on a general hospital ward but on the PACU.

24. Majewski W.D. An analysis of selected vital function parameters in patients undergoing anesthesia for laparoscopic and open prostatectomy. Surgical Endoscopy and Other Interventional Techniques 2014;28(SUPPL1):S173
Reason not included= study performed outside the general hospital ward

25. Maki H, Ogawa H, Tsukamoto S, Yonezawa Y, Caldwell WM. A system for monitoring cardiac vibration, respiration, and body movement in bed using an infrared. Conf Proc IEEE Eng Med Biol Soc 2010:5197-200.
Reason not included= no full text available. Author request sent by email without any reply.

26. Maniscalco M, Zedda A, Faraone S, Carratu P, Sofia M. Evaluation of a transcutaneous carbon dioxide monitor in severe obesity. Intensive Care Med 2008;34(7):1340-4.
Reason not included= study performed outside the general hospital ward

27. Mestek M., Addison P., Neitenbach A.-M., Bergese S., Kelley S. Accuracy of continuous noninvasive respiratory rate derived from pulse oximetry in congestive heart failure patients. Chest 2012;142(4)
Reason not included= not an original article

28. Mestek M.L., Ochs J.P., Addison P.S., Neitenbach A.-M., Bergese S.D., Kelley S.D. Accuracy of continuous non-invasive respiratory rate derived from pulse oximetry in patients with high respiratory rates. Anesthesia and Analgesia 2013 117 SUPPL. 1 (49-)
Reason not included= not an original article, study was partly performed in patients on the emergency department.

29. Mimoz O, Benard T, Gaucher A, Frasca D, Debaene B. Accuracy of respiratory rate monitoring using a non-invasive acoustic method after general anaesthesia. British Journal of Anaesthesia 2012 108:5 (872-875)
Reason not included= study performed outside the general hospital ward

30. Miyamoto Y, Yonezawa Y, Maki H, Ogawa H, Hahn AW, Caldwell WM. A system for monitoring heart pulse, respiration and posture in bed. Biomed Sci Instrum 2002;38:135-8. *Reason not included= no full text available.*

31. Mohr N.M., Wessman B. Continuous capnography should be used for every emergency department procedural sedation. Annals of Emergency Medicine 2013 61:6 (697-698)
Reason not included= study performed outside the general hospital ward

32. Morel G.L., Mahul P., Reche M., Viale J.P., Auboyer C., Geyssant A., Roche F., Barthelemy J.C., Pichot V. Real-time diaphragmatic electromyogram denoising using wavelet in intensive care unit and anesthesia. Fundamental and Clinical Pharmacology 2013 27 SUPPL. 1 (86-)
Reason not included= study performed outside the general hospital ward

33. Muders T, Luepschen H, Putensen C. Impedance tomography as a new monitoring technique. Curr Opin Crit Care 2010;16(3):269-75.
Reason not included= study performed outside the general hospital ward

34. Nikkola EM, Leino KA, Takala RSK, Kirvela OA, Salonen MAO. The validity of the static-charge-sensitive bed in the detection of fentanyl-induced respiratory depression. Acta Anaesthesiol Scand 2004;48(3):371-6.
Reason not included= study performed outside the general hospital ward

35. Orphanidou C, Clifton D, Khan S, Smith M, Feldmar J, Tarassenko L. Telemetry-based vital sign monitoring for ambulatory hospital patients. Conf Proc IEEE Eng Med Biol Soc 2009:4650-3.
Reason not included= not an original article, the study was performed outside the general hospital ward

36. Overdyk FJ, Carter R, Maddox RR, Callura J, Herrin AE, Henriquez C. Continuous oximetry/capnometry monitoring reveals frequent desaturation and bradypnea during patient-controlled analgesia. Anesth Analg 2007;105(2):412-8.
Reason not included= outcome was not defined as abnormal vital signs/SAE

37. Orr J, Long C, Brewer LA CO(2) waveform generator use in evaluating capnometer performance using previously recorded clinical data. Anesthesia and Analgesia 2012;115(2):S11
Reason not included= study performed outside the general hospital ward, and outcome was not defined as abnormal vital signs/SAE.

38. Palace ZJ, Mendelson G, Thaler MF, Margel A. The effect of a continuous patient monitoring system on reducing hospitalizations in the nursing home. Journal of the American Geriatrics Society 2013;61(SUPPL1):S68
Reason not included= study performed outside the general hospital ward

39. Pinna GD, Robbi E, Pizza F, Taurino AE, Pronzato C, La Rovere MT, Maestri R. Comparison between type 2 and type 3 portable monitoring in the assessment of sleep-disordered breathing in heart failure patients. European Journal of Heart Failure 2012;11(SUPPL1):S30
Reason not included= outcome was not defined as abnormal vital signs/SAE

40. Pedersen T, Hovhannisyan K, Møller AM, Smith AF, Lewsi SR. Pulse oximetry for perioperative monitoring. Cochrane Library 2009.
Reason not included= study performed outside the general hospital ward

41. Rowan C, Ahmed S, Hedlund T., Speicher R. Continuous capnography decreases the utilization of blood gases. Critical Care Medicine 2011;39(SUPPL12):91
Reason not included= study performed outside the general hospital ward

42. Ruiz-Rodriguez JC, Ruiz-Sanmartin A, Ribas V, Caballero J, Garcia-Roche A, Riera J, et al. Innovative continuous non-invasive cuffless blood pressure monitoring based on photoplethysmography technology. Intensive Care Medicine 2013;39(9):1618-25.
Reason not included= study performed outside the general hospital ward

43. Slight SP, Franz C, Olugbile M, Brown HV, Bates DW, Zimlichman E. Cost savings attributable to a continuous monitoring system in a medical-surgical unit. Journal of General Internal Medicine 2013;28(SUPPL1):S54
Reason not included= not an original article, as the data used was from a before-and after controlled study described in another (selected) paper.

44. Soto RG, Davis M, Faulkner MJ. A comparison of the incidence of hypercapnea in non-obese and morbidly obese peri-operative patients using the SenTec transcutaneous pCO(2) monitor. Journal of Clinical Monitoring and Computing 2014;28(3):293-8
Reason not included= outcome was not defined as abnormal vital signs/SAE

45. Srinivasa V, Kodali BS. Capnometry in the spontaneously breathing patient. Curr Opin Anaesthesiol 2004;17(6):517-20.
Reason not included= not an original article

46. Taenzer AH, Pyke J, Herrick MD, Dodds TM, McGrath SP. A comparison of oxygen saturation data in inpatients with low oxygen saturation using automated continuous monitoring and intermittent manual data charting. Anesth Analg 2014;
Reason not included= outcome was not defined as abnormal vital signs/SAE

47. Uemura K, Hayashi H, Tsubaki K, Egawa J, Inoue S, Furuya H, Kawaguchi M. Use of acoustic respiratory rate monitoring equipment Rad-87 in patients undergoing awake craniotomy. Journal of Neurosurgical Anesthesiology 2013;25(4):508
Reason not included= study performed outside the general hospital ward

48. Voepel-Lewis T, Parker ML, Burke CN, Hemberg J, Perlin L, Kai S, Ramachandran SK. Pulse oximetry desaturation alarms on a general postoperative adult unit: a prospective observational study of nurse response time. Int J Nurs Stud 2013;
Reason not included= outcome was not defined as abnormal vital signs/SAE

49. Voscopoulos C., Freeman J., Ladd D., Brayanov J.B., MacNabb C., George E. Use of a non-invasive respiratory volume monitor to identify opioid induced respiratory depression in the postanesthesia care unit. Anesthesia and Analgesia 2013;116(SUPPL1)
Reason not included= study performed outside the general hospital ward

50. Waldschmitt E, Smith H, Abosaida A. In-clinic evaluation of home respiratory equipment and treatment adherence in cystic fibrosis patients. Pediatric Pulmonology 2014;49(SUPPL38):358-9
Reason not included= study performed outside the general hospital ward
