# Supplementary material for: Non-Invasive Continuous Respiratory Monitoring on General Hospital Wards: A Systematic Review
Source: PLoS One. 2015 Dec 14;10(12):e0144626. doi: 10.1371/journal.pone.0144626 (PMC4684230; doi:10.1371/journal.pone.0144626)
Supplement: S1 Table — (DOCX) [file pone.0144626.s006.docx]

**S1 Table. Technical characteristics of non-invasive continuous monitors in diagnostic studies.**

|  |  | **Sensor technology** | | | | | **Signal analysis** | **Caregiver notification** |
| --- | --- | --- | --- | --- | --- | --- | --- | --- |
|  |  |  |  | **Additional physiological parameters monitored** | **Remarks** | |  |  |
| **Authors** | **Device** | **Respiratory parameters monitored** | **Sensing principle** |  | **Signal transmission** | **Detection of upper airway obstruction** | **Smart alarm control** | **Alarms displayed** |
| Anderson(22) | Monitron belt | BR | Chest wall movement through stretch | HR | Wireless | - | - | B, NB |
| Flisberg(23) | Optovent nasal probe | BR | Water vapor sensing in expired air | - | Hard-wired | + | - | B |
| Hravnak(24) | Biosign algorithm | BR, SpO2 | Chest wall impedance, pulse oximetry | HR, BP | Hard-wired | - | + | (Disabled) B, C |
| Jacobs(25) | Passive sensor array | BR | Chest wall movement through stretch | HR | Wireless | - | - | (Disabled) NB |
| Zimlichman(26) | Early Sense piezoelectric sensor | BR | Chest wall movement through stretch | HR | Wireless | - | +/- | (Disabled) B, C, NB |

Hemoglobin oxygen saturation (SpO2), Breathing rate (BR), Heart rate (HR), Blood pressure (BP), Temperature (T), Nurse beeper (NB), Bedside (B), Central nursing station (C).

Smart alarm control: (-) none, (+/-) efforts in data modeling techniques to improve interpretation, e.g. 24-hour trend analysis (+) advanced data modeling techniques as fuzzy logic, neural networking, and pattern recognition to ease interpretation.
