# Supplementary material for: Non-Invasive Continuous Respiratory Monitoring on General Hospital Wards: A Systematic Review
Source: PLoS One. 2015 Dec 14;10(12):e0144626. doi: 10.1371/journal.pone.0144626 (PMC4684230; doi:10.1371/journal.pone.0144626)
Supplement: S2 Table — (DOCX) [file pone.0144626.s007.docx]

**S2 Table. Summary of methodological quality of the diagnostic studies.**

| **Diagnostic studies** | Representative sample | Cohort study design | Adequate reference standard | Adequate index test description | Blinding of results | Complete verification with reference | Demographic characteristics | Report estimates of diagnostic accuracy | Withdrawals explained | Clinical applicability discussed |
| --- | --- | --- | --- | --- | --- | --- | --- | --- | --- | --- |
| Anderson(22) | + | - | - | + | - | - | - | - | - | - |
| Flisberg(23) | + | - | - | + | - | - | + | - | - | + |
| Hravnak(24) | + | + | - | - | + | - | + | - | + | + |
| Jacobs(25) | + | + | + | - | + | + | - | + | + | + |
| Zimlichman(26) | - | - | +/- | + | - | +/- | + | + | - | + |

Diagnostic research assesses whether a diagnostic test is able to discriminate between patients with or without the disease. In this particular setting, the diagnostic challenge was to distinguish between normal and abnormal vital signs or ‘vital stability’ versus ‘vital instability’, repeatedly at various time points during general hospital ward admission. Four diagnostic studies had a cross-sectional design.(22-25) In contrast with the index monitor (i.e., the respiratory monitor under research), that typically stored data continuously (i.e., at a very high frequency), the reference measurements were performed at discrete time points in three of these studies.(22-24) *Anderson* used an observer that verified alarms (i.e., abnormal vital signs by the index test), and *Flisberg* checked periods of abnormal breathing as indicated by the index monitor with blood gas analysis.(22,23) In both studies the reference standard was unable or too insensitive to detect true periods with abnormal breathing. This affects the interpretation of the study results, as the number of false negatives for the index monitor is probably underestimated. The *Hravnak 2008* study differs from the other diagnostic studies because the index monitor used similar hardwired sensor technology as the reference test.(24) Their reference standard includes Medical Emergency Team (MET) activation criteria based on the continuous sensor technology and nurse observations. *Jacobs* used a reference standard which also continuously measured physiological parameters, a conventional continuous monitor used by default in high care facilities(25),which allowed the authors to present estimates for diagnostic accuracy and precision. However, reference data was not completed due to discomfort of ECG leads. *Zimlichman* largely followed a diagnostic approach, but the study was not cross-sectional.(26) The monitor was used to distinguish between patients with or without the occurrence of a critical event (ICU transfer, intubated and mechanically ventilated, or cardiac arrest) within 24 hours after a vital sign measurement. Hence, the reference standard did not measure vital stability and –instability at that moment in time, but the reference diagnosis was made by follow-up.
