# Supplementary material for: Non-Invasive Continuous Respiratory Monitoring on General Hospital Wards: A Systematic Review
Source: PLoS One. 2015 Dec 14;10(12):e0144626. doi: 10.1371/journal.pone.0144626 (PMC4684230; doi:10.1371/journal.pone.0144626)
Supplement: S3 Table — (DOCX) [file pone.0144626.s008.docx]

**S3 Table. Summary of characteristics and outcomes for diagnostic studies**.

| **Authors** | **Year** | **No. of patients** | **Mean age (year)** | **Ward** | **Nurse: patient ratio** | **Monitoring intensity *** | **Outcome** | **Outcome measure** | **Patient comfort †** | **Alarm/ False alarm rate** |
| --- | --- | --- | --- | --- | --- | --- | --- | --- | --- | --- |
| *Diagnostic studies* | | | | | | | | | | |
| **Anderson**(22) | 1992 | 8 | 71 | Surgical | ? | Conditional | Bradypnea, apnea | Not extractable | - | - |
| **Flisberg**(23) | 2002 | 15 | 62 | Surgical | ? | Conditional | Bradypnea | PPV= 21.4%‡ | - | - |
| **Hravnak**(24) | 2008 | 326 | 58 | Surgical | 1:4-6 | Surveillance | Vital instability | PPV= 59.4%‡ | - | 0.54 alerts per patient day‡ |
| **Jacobs**(25) | 2007 | 287 | 48 | Medical Surgical | ? | Surveillance | Vital instability | PPV= 98.9%  Sens= 63.1% | + | 0.16 false alerts per patient day |
| **Zimlichman**(26) | 2011 | 113 | 69 | Medical | ? | Conditional | Critical events | PPV= 50% §  Sens= 55% §  AUC= 0.75(p<.001) § | + | 2.7 alerts per patient day |

* Monitoring intensity: Defined as surveillance when monitored for 100% during their entire hospitalization, or conditional in case of specific patient categories for a limited period.

† Patient comfort: (+) good patient comfort as derived from the study results or reviewers’ rating.

‡ Outcome measures and alarm rates are calculated with extracted information from the paper.

§ Statistics computed for detection of critical events with respiratory and heart rate alerts with retrospective (post hoc) determined optimal thresholds.

Sens= sensitivity, PPV = positive predictive value, AUC= area under the ROC (receiver operating characteristic) curve.

The *Hravnak* intervention study(17) used the patients from the previously published diagnostic study(24) as the reference (before) population.
